# Role of Early PET/CT Imaging with 68Ga-PSMA in Staging and Restaging of Prostate Cancer

**DOI:** 10.1038/s41598-020-59296-6

**Published:** 2020-02-17

**Authors:** Andrew Barakat, Basel Yacoub, Maria El Homsi, Amro Saad Aldine, Albert El Hajj, Mohamad B. Haidar

**Affiliations:** 1 Department of Diagnostic Radiology, American University of Beirut Medical Center, Beirut, Lebanon; 2 Department of Radiology and Radiological Science, Medical University of South Carolina, Charleston, South Carolina United States; 3 Department of Radiology, University of Iowa, Iowa City, Iowa United States; 4 Department of Surgery, American University of Beirut Medical Center, Beirut, Lebanon

**Keywords:** Cancer imaging, Prostate cancer, Prostate cancer, Prostate

## Abstract

Ga-68 Prostate-Specific Membrane Antigen PET/CT is a new tool for the assessment of prostate cancer. Standard imaging time is 60 minutes post injection of radiotracer. At 60 minutes, there is physiologic accumulation of radiotracer in the urinary bladder which may cause some lesions in its vicinity to be obscured. Our aim is to determine if early imaging at 3 minutes in addition to standard imaging at 60 minutes can improve the detection of PSMA-avid lesions. A retrospective review of 167 consecutive patients was conducted. Overall, 115 patients (68.9%) were ruled to have prostate cancer based on imaging as seen on early or standard PET/CT images. In 106/115 (64%), the lesions were detected on both early and standard imaging; in 8/115 (6.9%), the lesions were only detected on early imaging; in 1/115 (0.6%) the lesion was detected only on standard imaging. The addition of early imaging significantly improved the overall detection rate of PSMA-avid lesions (p = 0.039). The ratio of patients with lesions detected on early imaging but not on standard imaging in restaging group was 7/88 and was higher than that in staging group 1/79 (p = 0.043). We recommend early imaging in addition to the standard imaging in Ga-68 PSMA PET/CT, particularly in patients presenting for restaging of prostate cancer.

## Introduction

Prostate cancer is the fourth most common cancer globally with an estimated incidence of 1.6 million cases per year, leading to an estimated 366 thousand deaths^1^. It is also the cause of the second most common cancer associated mortality in the United States, second to lung and bronchial cancers, and just ahead of colorectal cancer^2^. The standard protocol for treatment of prostate cancer most often involves prostatectomy or radiotherapy. Nonetheless, biochemical recurrence as defined by an increase in post-therapy PSA level has been found in up to 28% of patients at 5 years post radical prostatectomy^3–6^. With such substantial burden of disease, development of newer and more accurate imaging techniques and modalities for prostate cancer is of paramount importance to allow for more accurate staging and restaging of the disease.

Imaging modalities for prostate cancer primary disease, recurrence or metastasis have traditionally included CT, MRI and bone scintigraphy. Newer modalities for diagnosis and staging have also been used. These include PET scan with fluorodeoxyglucose (FDG) or choline based radiotracers. A newer class of radiotracers of great potential in target imaging of prostate cancer are prostate specific membrane antigen (PSMA) inhibitors. They target PSMA, a transmembrane protein whose expression is increased up to 1000-folds in prostatic cancer cells^7^. PSMA inhibitors are most commonly coupled with 68-Gallium isotope and used in the radiotracer 68Ga-PSMA-HBED-CC for PET/CT and PET/MRI.

The role of PET/CT imaging with 68Ga-PSMA is being evaluated in staging and restaging of prostate cancer. Studies by Sachpekidis *et al*. have demonstrated an overall detection rate of 96% in patients with primary prostate cancer and 71% in patients with biochemical recurrence. They also demonstrated a positive correlation between SUVmax and SUVavg values of PSMA-avid lesions with PSA levels and Gleason scores, leading to increased lesion detection rates in patients with higher PSA levels^8,9^. In a meta-analysis on sixteen studies involving a total of 1309 patients by Perera *et al*., the sensitivity and specificity of 68Ga-PSMA-11 PET/CT were reported at 80% and 97% respectively based on a per-lesion analysis^10^.

The current standard practice for imaging time in PET/CT for prostate cancer is 60 minutes post injection (p.i) of 68Ga-PSMA radiotracer^11^. This is supported by the joint guideline published by Society of Nuclear Medicine and Molecular Imaging (SNMMI) and The European Association of Nuclear Medicine (EANM) which recommends imaging at 60 minutes to allow for radiotracer uptake. An acceptable imaging time, according to the guideline, ranges from 50 to 100 minutes p.i with the possibility of imaging at 3 hours post injection. This late imaging may improve detection of lesions with low PSMA expression and slower accumulation of the radiotracer and it may also improve detection of lesions in close proximity to the ureter or the bladder. The guideline also recommends voiding immediately prior to image acquisition in an attempt to reduce physiologic activity of the radiotracer in the urinary system with the possibility of administering of furosemide before or after the injection of the radiotracer^12^.

Studies have demonstrated that during the first few minutes post injection, local prostate cancer lesions show radiotracer uptake occurring before its accumulation in the urinary bladder^13^. Thus, early imaging with PET/CT in the first few minutes p.i, in addition to imaging at 60 minutes p.i, may improve detection of PSMA-avid lesions in proximity to the urinary bladder which may otherwise have been obscured by physiologic activity in the bladder. The aim of our study is to show that imaging in the first 3 minutes p.i in addition to imaging at 60 minutes p.i increases detection rates of suspected prostate cancer lesions.

## Materials and Methods

### Ethical approval

This study was approved by the Institutional Review Board (IRB) at the American University of Beirut Medical Center under the protocol BIO-2017-0386. The need for written informed consent was waived by IRB because of the study’s retrospective nature and involvement of no more than minimal risk to patients. All procedures performed in this study were conducted in accordance with the tenets of the Declaration of Helsinki and its amendments.

### Patients

167 consecutive patients who had undergone both early and standard 68Ga-PSMA-11 PET/CT imaging between December 2016 and July 2017 were retrospectively analyzed. All the patients were referred for imaging for either staging or restaging of prostate cancer.

### Radiotracer

Good Manufacturing Practice (GMP) certified 68-Gallium Radiolabelling Kit by Isotope Technologies Garching (Munich, Germany) and single-use sterile cassette by ABX Advanced Biochemical Compounds (ABX) (Radeberg, Germany) were labeled to 10 μg PSMA-11.

### Image acquisition

Each patient received 65–178 MBq (mean 113.3 ± 21.2MBq) of 68Ga-PSMA-11 intravenously while on the PET/CT scanner. Thereafter, two sets of PET/CT images were obtained in supine position from each patient. The early set was for the pelvic region only and was acquired starting with injection and for 3 minutes p.i. The standard set was for the whole body and was acquired at 60 minutes p.i. as per the current accepted guidelines. Patients were asked to urinate before the second set.

PET/CT acquisition was performed on a Philips Gemini TF 16 PET/CT scanner. This system is time-of-flight (TOF) capable and has full three-dimensional PET capabilities together with a 16-slice Brilliance-CT. PET acquisition time was 1.5–2 minutes per bed position for the body and 0.5–1 minute per bed position for the lower extremities using the TOF-PET technique. The exact CT parameters used for unenhanced acquisition were: collimation, 16 × 1.5 mm; pitch, 0.8; rotation time, 0.75 sec; effective tube current-time product is based on a scale that is dependent on patient weight; tube voltage of 140 kVp. Effective tube current-time scale is as follows: 35 mA for patients below 60 kg, 50 mA for patients between 60–80 kg, and 65 mA for patients between 80–100 kg. Based on the body mass index of patients over 100 kg, a range of 65–300 mA is applied. Finally, image reconstruction was performed at a slice thickness of 5 mm, and 2 mm for PET/CT.

### Image evaluation

Images were interpreted with the dedicated commercially available software IntelliSpace Portal 8.0 by Philips Healthcare (Amsterdam, Netherlands) which allows the review of PET, CT and fused imaging data in axial, coronal, and sagittal planes. A nuclear medicine physician and a radiology resident, with at least 5 years and 6 months of experience respectively in reading 68Ga-PSMA-11 PET/CT scans, independently reviewed the early and standard images then qualitatively and quantitatively scored the visibility of prostate cancer lesions. All disagreements in reading the images were later resolved by consensus. Both readers were blinded to the patients’ clinical information. Any focal uptake in prostate or prostate bed with SUVmax greater than 2 and that did not correspond to physiologic radiotracer accumulation was considered pathologic and suggestive of malignancy. In our study, such lesions were deemed as ‘positive’ while lesions that did not show increased tracer uptake compared to surrounding tissue was deemed as ‘negative’.

### Statistical analysis

Age was reported as mean ± SD. PSA and SUVmax were reported as median with interquartile range. Statistical analysis was performed using SPSS version 25.0 (IBM Corp., Armonk, NY, USA). Detection rates among early and standard imaging were compared using the McNemar test. Linear correlation between continuous variables was assessed using Pearson Correlation Coefficient. Difference in proportions was assessed using Two Proportion Z-Test. A p-value lower than 0.05 was considered significant.

## Results

Of the 167 patients, 79 were presenting for initial staging and 88 for restaging. 79 patients had been referred to PET/CT for staging before initiating any treatment. 40 had undergone prostatectomy only, 18 had received hormonal therapy only, 4 had received radiotherapy only and 26 had received two or more of the previous treatments. The mean age of the patients was 69.0 years (SD = 8.7). Median PSA level was 7.05 ng/ml (Q1 = 2.43, Q3 = 21.75) (Table 1).

**Table 1:** Patient characteristics.

Continuous variables		Mean	Standard Deviation
Age (years)		69.00	8.74
		**Median**	**IQR**
PSA level* (ng/ml)		7.05	(2.43–21.75)
**Categorical variables**		**Count**	**Percentage**
Indication	Staging	79	47.3
	Restaging	88	52.7
Treatment	Radiotherapy only	3	1.8
	Hormonal therapy only	14	8.4
	Prostatectomy only	32	19.2
	Radiotherapy and hormonal therapy	15	9.0
	Radiotherapy and prostatectomy	10	6.0
	Hormonal therapy and prostatectomy	6	3.6
	Radiotherapy, hormonal therapy and prostatectomy	8	4.8
	Before any treatment	79	47.3

^*^In 11 patients, PSA level could not be obtained from records.

Of the included 167 patients, 115 (68.9%) were found to have one or more radiotracer avid lesions within the prostate gland or bed on either early or standard imaging. In 114 of the 167 patients (68.3%), the lesions were seen on early imaging. In 107 of 167 (64.0%), the lesions were seen on standard imaging. The higher detection rate on early imaging was statistically significant on McNemar’s test (p = 0.039). In 8/167 (4.8%), the lesion was only seen on early imaging. In 1/167(0.6%), the lesion was only seen on standard imaging. In 52/167 (31.1%), no lesions were detected in neither early nor standard imaging (Table 2).

**Table 2:** Number of lesions detected on early and standard Ga-68 PSMA PET/CT.

		Standard imaging		
		**+**	**−**	**Total**
**Early Imaging**	**+**	106	8	114
	**−**	1	52	53
	**Total**	107	60	167

Among the staging group, 75 out of 79 patients (94.9%) had positive uptake on either early or standard imaging. 75 out of 79 patients (94.9%) were positive on early imaging, of whom 74 were positive on both early and standard imaging and 1 was only positive on early imaging. (Table 3) Among the restaging group, 40 of 88 patients (45.5%) had positive uptake on either early or standard imaging, of whom 32 were positive on both early and standard imaging, 7 were only positive on early imaging and 1 patient was only positive on standard imaging. (Table 4) Therefore, in the staging group, 1 out of 79 patients had lesions detected only on early imaging but not on standard imaging. In comparison, this ratio was 7 out of the 88 patients in the restaging group and was significantly higher than that in the staging group. (p = 0.043).

**Table 3:** Number of patients from the staging group with lesions detected on early and standard Ga-68 PSMA PET/CT.

		Standard imaging		
		**+**	**−**	**Total**
**Early Imaging**	**+**	74	1	75
	**−**	0	4	4
	**Total**	74	5	79

**Table 4:** Number of patients from the restaging group with lesions detected on early and standard Ga-68 PSMA PET/CT.

		Standard imaging		
		**+**	**−**	**Total**
**Early Imaging**	**+**	32	7	39
	**−**	1	48	49
	**Total**	33	55	88

In the staging group, the median PSA level was 16.1 ng/ml (Q1 = 6.90, Q3 = 49.25), median SUVmax on early imaging was 6.7 (Q1 = 4.2, Q3 = 10.3) and median SUVmax on standard imaging was 8.2 (Q1 = 4.3, Q3 = 16.9). In the restaging group the median PSA level was 6.24 ng/ml (Q1 = 3.09, Q3 = 22.93), median SUVmax on early imaging was 6.3 (Q1 = 4.1, Q3 = 10.6) and median SUVmax on standard imaging was 7.7 (Q1 = 4.0, Q3 = 13.3).

There was a positive correlation between PSA value and SUVmax. When correlating PSA values and SUVmax obtained on early imaging, Pearson correlation factor r was 0.413 (p < 0.001). The relationship between PSA values and SUVmax obtained on standard imaging was similar with r = 0.408 (p < 0.001). When considering only patients presenting for staging, this correlation was stronger with r = 0.569 (p < 0.001) when comparing PSA values and SUVmax obtained on early imaging. While on standard imaging, a slightly weaker correlation r = 0.522 (p < 0.001) was obtained when comparing PSA values and SUVmax obtained in the same patients presenting for staging.

The median PSA level in patients with increased uptake on the early images (n = 114) was 13.00 ng/ml (Q1 = 5.40, Q3 = 38.70) with a median SUVmax of 6.5 (Q1 = 4.2, Q3 = 10.38). In those with increased uptake on the standard images (n = 107), median PSA level was 14.14 ng/ml (Q1 = 6.01, Q3 = 43.83) with a median SUVmax of 8.15 (Q1 = 4.30, Q3 = 14.63). While in patients with increased uptake on the early images and no uptake on the standard images (n = 8), the median PSA was 3.30 ng/ml (Q1 = 0.59, Q3 = 12.20) with a median SUVmax of 2.85 (Q1 = 2.08, Q3 = 8.88). Finally, in the patient with increased uptake on standard images but no uptake on early images (n = 1), PSA was 0.08 ng/ml and SUVmax on early and standard imaging were 2 and 6 respectively. When comparing the PSA levels of the 106 patients with increased uptake on both early and standard imaging (median PSA = 14.40 ng/ml) to the 8 patients with increased uptake only on early imaging (median PSA = 3.30 ng/ml), we found no statistically significant difference (p = 0.254).

## Discussion

**Figure 1:**
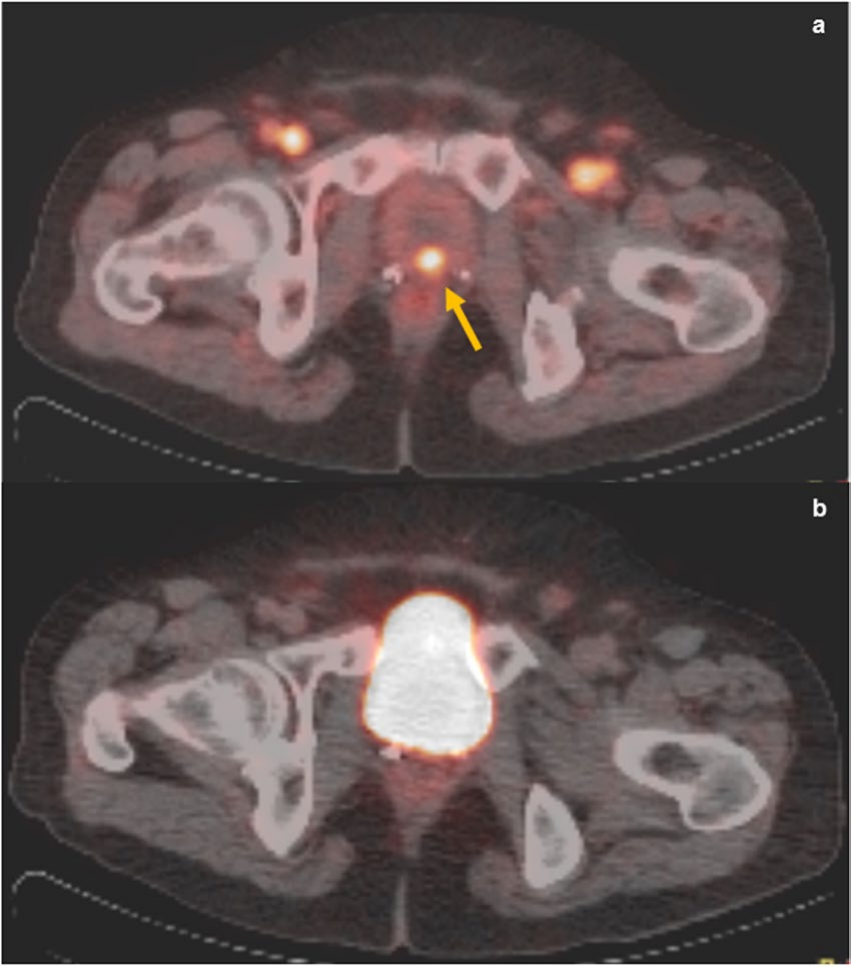
73 year old male presenting for prostate cancer staging. (**a**) The prostate gland shows a focus of increased activity in its center (arrow) with SUV max 4.2 at 3 minutes. Radiotracer activity in the inguinal vessels is also visible. Early PET/CT imaging helped detect tumor lesion that was obscured by radiotracer activity in the bladder on standard imaging at 60 minutes post-injection (**b**).

While the current standard of practice recommends imaging at 60 minutes post injection of 68 Ga-PSMA in PET/CT for prostate cancer, only a few studies have discussed the usefulness of imaging at an earlier time^12^. The rationale behind the use of early imaging is that cancer lesions show radiotracer uptake occurring before radiotracer accumulation in the urinary bladder, which may otherwise mask these lesions during the standard imaging at 60 minutes. Uprimny *et al*. found that bladder activity was absent within the first 3 minutes post injection, with all pathologic lesions showing radiotracer uptake at 3 minutes^14^. In a follow-up study performed using early imaging on 203 patients with biochemical recurrence, it was shown that early imaging significantly increased detection rate of local recurrence from 12.8% to 24.6% and that equivocal findings on imaging at 60 minutes significantly decreased from 15.8% to 4.5%^15^. Perveen *et al*. also prospectively studied 15 prostate cancer patients who underwent PSMA PET/CT, with all pathologic lesions showing early radiotracer uptake at 3 minutes and insignificant bladder activity as compared to standard imaging at 60 minutes. Similarly, in our study 8/167 (4.8%) had lesions seen on early imaging and masked by bladder activity on standard imaging. There was a statistically significant increase in detection rate from 64.0% using standard imaging to 68.3% when performing early imaging. Upon review of the images, the lesions that were missed on standard imaging were in the anterior aspect of the prostate or anterior aspect of the surgical bed in cases of prostatectomy. This demonstrates the importance of early imaging in enhancing target-to-background ratio of malignant prostatic lesions due to decreased radioactivity in the urinary bladder, thereby improving detection of lesions that would have been missed on standard imaging and thus changing the management of these patients. (Fig. 1).

Currently, many PSMA PET/CT protocols entail voiding before the scan or the administration of diuretics such as furosemide to wash out accumulated radiotracer from the bladder. This may improve the visualization of small prostatic lesions and surrounding lymph nodes during standard imaging. However, these methods are time consuming, do not entirely reduce bladder radioactivity, and also the use of diuretics is often avoided in patients with kidney disease^13^. Additionally, Perveen *et al*. showed that even after administration of diuretics prior to imaging at 60 min p.i, bladder activity remained higher than that during early imaging, with the latter modality providing better delineation of prostatic and regional lesions^16^.

The prostate gland normally expresses PSMA^17^, however cancerous cells have increased expression, thus enabling the detection of cancer lesions by 68Ga-PSMA-11^18^. Several studies performed showed a statistically significant correlation between PSA level and SUVmax in primary tumors^9,19,20^. One study performed on 90 patients further confirmed those results and showed correlation between SUVmax in the primary tumor as well as metastatic lymph nodes with PSA level^20^. Therefore, as PSA levels increases, the SUVmax values increase, and thus possibly enabling improved detection of lesions. This was reflected in several aspects of our results. First, we found a positive correlation between PSA levels and SUVmax values and this correlation was stronger in patients coming for staging. Second, patients who were undergoing PET/CT for restaging purposes and thus were more likely to have undergone surgical resection, hormonal therapy, radiotherapy or a combination of these, did in fact have lower median PSA levels than those undergoing staging and lower SUVmax on both early and standard imaging. Finally, when we stratified our patients into staging versus restaging groups, we found that out of the 8 patients who were positive on early but negative on standard imaging, 7 were from the restaging group, while only 1 was of the staging group. The difference between these two ratios (7/88 vs 1/79) was found to be statistically significant.

Our results are in accordance with those of Kabasakal *et al*., which demonstrated high sensitivity and specificity of PSMA PET/CT in patients with low PSA levels (between 0.2–5 ng/mL)^21^. Furthermore, we found that in patients with increased uptake on the early images and no uptake on the standard images, the median PSA level was 3.3 ng/ml, which is considered clinically low. These points allow us to conclude that early imaging at 3 minutes, in addition to improving the detection of cancerous prostate lesions, is particularly useful in patients undergoing restaging for biochemical recurrence and with low PSA levels.

As per Giesel *et al*., a cut off of SUVmax of 2 was used to consider a lesion positive on the early imaging. The only patient in our study who showed increased uptake on standard but not early imaging had a single lesion with an SUVmax of 2.0 on early imaging, and it was an equivocal finding. He was considered negative for the sake of analysis. The standard images confirmed the suspicion by demonstrating further increase in the SUVmax to a value of 6^22^.

## Strengths and Limitations

One limitation of this study was that there was no histological confirmation of the lesions. Our aim was only to compare detection of PSMA-avid lesions in early imaging using PSMA PET/CT to the current standard imaging at 60 minutes. Verification of positive findings for the purpose of calculating sensitivity and specificity of PET/CT was not performed given the aim of this study. With that in mind, future studies should be performed to correlate lesions found on early PET/CT with other imaging modalities and with pathology results.

## Conclusion

Early imaging with 68Ga-PSMA-11 PET/CT increases detection of PSMA-avid lesions, suggestive of prostate cancer or its recurrence, in the anterior transitional zone of the prostate gland or the anterior aspect of the prostate bed and thus changing the management of these patients. This increase in detection is especially important in patients presenting for restaging.

## Data Availability

The dataset generated and analyzed during the current study are available from the corresponding author on reasonable request.
